# Impact of CT Intensity and Contrast Variability on Deep-Learning-Based Lung-Nodule Detection: A Systematic Review of Preprocessing and Harmonization Strategies (2020–2025)

**DOI:** 10.3390/diagnostics16020201

**Published:** 2026-01-08

**Authors:** Saba Khan, Muhammad Nouman Noor, Imran Ashraf, Muhammad I. Masud, Mohammed Aman

**Affiliations:** 1 School of Computing, National University of Computer & Emerging Sciences (FAST-NUCES), Islamabad 44000, Pakistan; 2 Department of Electrical Engineering, College of Engineering, University of Business and Technology, Jeddah 21361, Saudi Arabia; 3 Department of Industrial Engineering, College of Engineering, University of Business and Technology, Jeddah 21361, Saudi Arabia

**Keywords:** lung cancer, computed tomography, Hounsfield Unit variability, deep learning, preprocessing, image harmonization, CLAHE, ComBat, transformer networks, systematic review

## Abstract

**Background/Objectives**: Lung cancer is the leading cause of cancer-related mortality worldwide, and early detection using low-dose computed tomography (LDCT) substantially improves survival outcomes. However, variations in CT acquisition and reconstruction parameters including Hounsfield Unit (HU) calibration, reconstruction kernels, slice thickness, radiation dose, and scanner vendor introduce significant intensity and contrast variability that undermine the robustness and generalizability of deep-learning (DL) systems. **Methods**: This systematic review followed PRISMA 2020 guidelines and searched PubMed, Scopus, IEEE Xplore, Web of Science, ACM Digital Library, and Google Scholar for studies published between 2020 and 2025. A total of 100 eligible studies were included. The review evaluated preprocessing and harmonization strategies aimed at mitigating CT intensity variability, including perceptual contrast enhancement, HU-preserving normalization, physics-informed harmonization, and DL-based reconstruction. **Results**: Perceptual methods such as contrast-limited adaptive histogram equalization (CLAHE) enhanced nodule conspicuity and reported sensitivity improvements ranging from 10 to 15% but frequently distorted HU values and reduced radiomic reproducibility. HU-preserving approaches including HU clipping, ComBat harmonization, kernel matching, and physics-informed denoising were the most effective, reducing cross-scanner performance degradation, specifically in terms of AUC or Dice score loss, to below 8% in several studies while maintaining quantitative integrity. Transformer and hybrid CNN–Transformer architectures demonstrated superior robustness to acquisition variability, with observed AUC values ranging from 0.90 to 0.92 compared with 0.85–0.88 for conventional CNN models. **Conclusions**: The evidence indicates that standardized HU-faithful preprocessing pipelines, harmonization-aware modeling, and multi-center external validation are essential for developing clinically reliable and vendor-agnostic AI systems for lung-cancer screening. However, the synthesis of results is constrained by the heterogeneous reporting of acquisition parameters across primary studies.

## 1. Introduction

Lung cancer remains one of the leading causes of cancer-related mortality globally, with more than two million new cases diagnosed annually [1]. Survival outcomes depend heavily on early detection, as five-year survival increases from below 15% in advanced stages to more than 60% when tumors are identified early [2,3]. Consequently, low-dose computed tomography (LDCT) has been established as the gold standard screening method for detecting small pulmonary nodules before symptoms appear [3]. However, LDCT introduces noise and intensity inconsistencies due to dose reduction, and these variations complicate both radiologist interpretation and automated analysis [4].

Computed tomography (CT) images are quantified using Hounsfield Units (HU), where reconstruction kernels, slice thickness, radiation dose, and vendor-specific algorithms strongly influence image texture, contrast, and noise patterns [5]. Even scans from the same patient may exhibit marked differences when acquired using different scanners or protocols, leading to shifts in intensity distributions and nodule appearance [6]. These variations create substantial challenges for artificial intelligence (AI) systems, which typically assume consistent input characteristics during training.

While LDCT remains the gold standard for structural lung screening, it is strictly limited to anatomical assessment and often lacks the functional specificity required for complex oncological cases. In scenarios such as cancer of unknown primary (CUP), standard anatomical imaging frequently fails to localize the lesion. In such contexts, complementary strategies like molecular imaging (e.g., PET/CT) have demonstrated superior detection rates of 38–83% compared with conventional methods. Similarly, MRI-based radiomics has shown promise in soft-tissue characterization by extracting quantitative texture features (e.g., GLCM inverse variance) that escape human perception, enabling precise differential diagnosis in complex head and neck tumors. Although this review focuses on CT-based deep learning, acknowledging these multimodal diagnostic pathways is critical for a holistic understanding of pulmonary and general oncology [7,8].

Deep-learning models including convolutional neural networks (CNNs), U-Net-based segmentation systems, and Transformer architectures have shown strong performances on benchmark datasets [9]. Yet, their accuracy decreases by 10–20% when exposed to unseen scanners, reconstruction kernels, or dose levels [10]. This domain shift increases false negatives for small or low-contrast nodules and reduces clinical reliability [11]. The issue is further compounded by the widespread use of enhancement techniques such as histogram equalization or CLAHE, which improve visual contrast but often distort HU values and undermine radiomic reproducibility [12]. In contrast, harmonization methods such as ComBat, kernel matching, and physics-informed denoising aim to standardize intensity distributions while preserving quantitative integrity [13].

Although publicly available datasets such as the Lung Image Database Consortium (LIDC-IDRI) and LUng Nodule Analysis 2016 (LUNA16) have facilitated algorithm development, they represent limited scanner diversity and do not reflect real-world multi-institutional variability [14]. As a result, AI models trained exclusively on such datasets may generalize poorly across clinical environments [15]. Improving robustness requires a structured understanding of how acquisition variability affects AI performance and which preprocessing and harmonization strategies effectively mitigate these effects.

This systematic review addresses these challenges by synthesizing evidence published between 2020 and 2025 on (1) how acquisition- and reconstruction-related variability impacts AI-based lung-nodule detection, (2) the effectiveness of preprocessing and harmonization strategies in managing intensity and contrast heterogeneity, and (3) the robustness of different deep-learning architectures under variable imaging conditions. The review further highlights dataset limitations, gaps in external validation practices, and the need for standardized, HU-faithful workflows to support the clinically reliable deployment of AI-assisted lung-cancer screening.

To date, however, existing reviews remain fragmented in their treatment of CT acquisition variability and provide limited insight into how reconstruction kernels, slice thickness, radiation dose, vendor-specific reconstruction algorithms, and HU calibration inconsistencies collectively influence the robustness of AI-based lung-cancer detection systems. Most prior surveys focus primarily on model architectures or overall diagnostic performance, offering little systematic comparison of HU-preserving preprocessing techniques, perceptual enhancement methods, physics-informed harmonization strategies, and deep-learning-based reconstruction approaches within a unified framework. The limited adoption of multi-center external validation and the widespread absence of standardized reporting for essential acquisition parameters further obscure the true generalizability of published AI systems. These gaps underscore the need for a comprehensive, methodologically focused synthesis that directly examines how acquisition variability and preprocessing workflows influence model stability, reproducibility, and clinical applicability.

Addressing these limitations, the present systematic review contributes a consolidated and quantitative assessment of the field by mapping the impact of CT acquisition variability including differences in reconstruction kernels, slice thicknesses, dose categories, and vendor-specific characteristics on segmentation, detection, and malignancy classification performance [16,17] across 100 studies published between 2020 and 2025. It provides a comparative evaluation of preprocessing and harmonization techniques, clearly distinguishing HU-faithful normalization approaches from perceptual contrast-enhancement methods and examining their effects on AUC, Dice scores, radiomic stability, and cross-scanner generalization. The review further analyzes the robustness of modern AI architectures, including CNNs [18,19], attention-based networks, Transformers, hybrid segmentation–classification pipelines, and radiomics deep-learning fusion models under heterogeneous imaging conditions. Finally, it identifies key methodological gaps such as inconsistently reported acquisition metadata, the lack of standardized preprocessing pipelines, minimal external validation, and risks associated with generative adversarial networks (GANs)-based harmonization and outlines practical recommendations for building clinically reliable, vendor-agnostic CT-based AI workflows. Together, these contributions provide a cohesive and rigorous foundation for advancing robust, reproducible, and clinically deployable AI systems for lung-cancer screening.

## 2. Methodology

**Figure 1:**
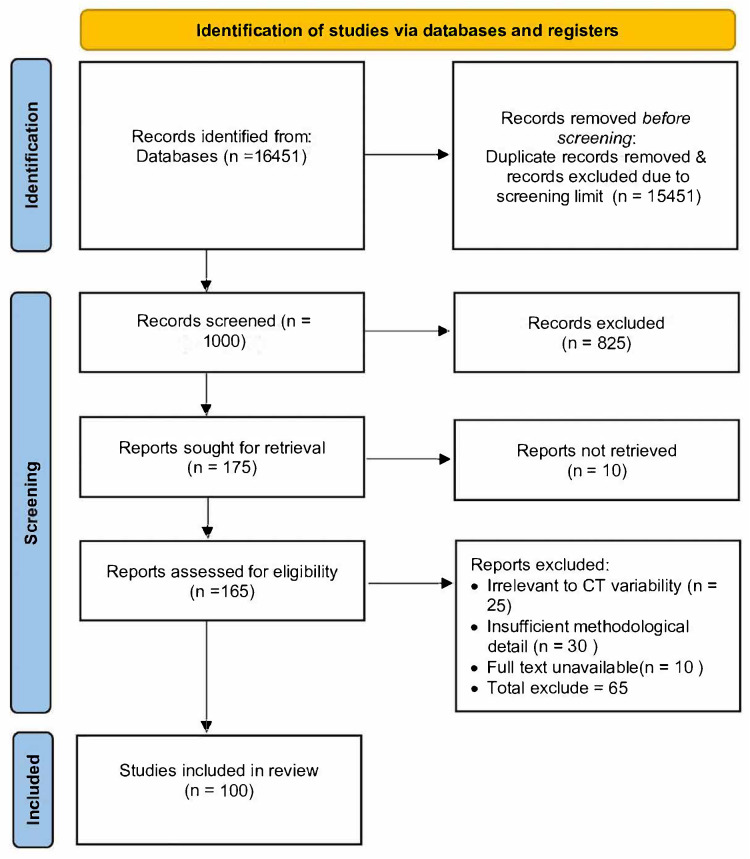
PRISMA 2020 flow diagram illustrating the identification, screening, eligibility, and inclusion of studies. This PRISMA diagram is adapted from the PRISMA 2020 statement [20].

This systematic review was conducted in full accordance with the PRISMA 2020 guidelines [20], and the reporting follows all items outlined in the PRISMA checklist. The review protocol was not preregistered in PROSPERO or any other registry; however, all methodological steps including search strategy, screening, eligibility assessment, data extraction, and synthesis were performed following PRISMA standards to ensure transparency and reproducibility. A completed PRISMA checklist is provided in the Supplementary Materials, and the PRISMA flow diagram summarizing identification, screening, eligibility, and inclusion is presented in Figure 1.

### 2.1. Research Questions and Objectives

The review was designed to address key research questions (RQs) regarding the impact of CT variability on AI performance and to establish corresponding objectives for synthesis. Specifically, the study sought to answer:

- RQ1: How do variations in CT acquisition parameters (e.g., kernel, dose, vendor) affect AI diagnostic performance?
- RQ2: Which preprocessing and harmonization methods effectively reduce intensity variability while preserving HU fidelity?
- RQ3: Which deep-learning architectures show the greatest robustness to cross-scanner variability?
- RQ4: How representative are widely used CT datasets in terms of scanner and dose diversity?
- RQ5: What methodological limitations exist in reporting acquisition parameters, dataset composition, preprocessing, and robustness evaluation?
- RQ6: To what extent do studies include external or multi-center validation, and how does this influence reported generalizability?

Correspondingly, the primary objectives were to quantify the impact of these variations, evaluate the efficacy of preprocessing strategies, identify robust architectures, and propose improvements for standardized reporting and multi-center validation.

### 2.2. Search Strategy

A systematic search was conducted across PubMed, IEEE Xplore, Scopus, Web of Science, and ACM Digital Library for the literature published from 2020 to 2025. To broaden coverage, the first 500 Google Scholar results were also screened manually, with irrelevant items (patents, theses, duplicates) removed. Although a preregistered protocol was not utilized, this review strictly adhered to all methodological procedures outlined in the PRISMA 2020 guidelines.

The search strategy was structured using a PICOC-style logic, focusing on CT acquisition variability [6], AI-based detection, and diagnostic performance. Table 1 outlines the PICOC components and the corresponding terminology used during query formulation.

To operationalize the PICOC framework, the search queries combined controlled vocabulary and free-text keywords relating to CT imaging, acquisition variability, preprocessing methods, and AI-based lung-cancer detection. Boolean operators ensured both sensitivity and precision across databases.

Search Queries Used:

1. (“lung cancer” OR “pulmonary nodule”) AND (“computed tomography” OR “CT” OR “LDCT”) AND (“Hounsfield” OR “reconstruction kernel” OR “slice thickness”OR “vendor” OR “radiation dose”) AND (“deep learning” OR “CNN” OR “Transformer” OR “AI detection”)
2. (“CT variability” OR “intensity harmonization” OR “ComBat” OR “kernel matching” OR “HU normalization”) AND (“lung nodule detection”) AND (“classification” OR “segmentation”)

Database-specific filters were applied to restrict searches to titles, abstracts, and metadata where possible. Google Scholar results were manually curated to exclude non-peer-reviewed sources and redundant citations.

### 2.3. Search Outcomes

The combined search retrieved 16,451 records, including 15,900 from Google Scholar. Given the large volume of results in Google Scholar, only the top 500 most relevant records were screened, while the remaining records were excluded due to the screening threshold. After removing duplicates and applying this threshold, 1000 unique records underwent title and abstract screening. Of these, 825 were excluded due to irrelevance (non-CT imaging, non-AI, or non-peer-reviewed sources). Full texts of 175 studies were assessed for eligibility, and 100 met all inclusion criteria (Table 2).

The complete workflow is illustrated in the PRISMA 2020 flow diagram (Figure 1), which visualizes all stages of identification, screening, eligibility, and inclusion.

This diagram confirms that the selection pipeline adhered strictly to PRISMA standards, showing transparent tracking of excluded studies and reasons for exclusion.

### 2.4. Eligibility Criteria and Study Selection Process

Eligibility was defined according to PRISMA guidelines to ensure a focused analysis of CT variability. Studies were included if they were peer-reviewed, published in English between 2020 and 2025, and explicitly addressed CT-acquisition variability in AI-based lung-nodule analysis. Gray literature and studies missing essential methodological details were excluded to maintain quality standards. The detailed inclusion and exclusion criteria utilized for screening are summarized in Table 3.

Applying these criteria, the study selection process was executed through a structured multi-stage workflow consistent with PRISMA 2020 standards:

- Title & Abstract Screening: Initial removal of studies unrelated to CT, AI, or imaging variability to filter out the irrelevant literature.
- Full-Text Review: A detailed assessment of the remaining articles to verify the reporting of acquisition parameters, model transparency, and performance metrics.
- Backward & Forward Reference Checking: A final supplementary search was conducted to ensure no relevant studies were missed beyond the initial database pool.

### 2.5. Data Extraction

A standardized data-extraction form was developed to ensure consistency and reproducibility across all included studies. To address the reviewers’ comments regarding quality assurance, the data extraction process was structured as follows: The primary reviewer (S.K.) extracted all relevant data fields from the 100 included studies. Subsequently, a second reviewer (M.N.N.) performed an independent validation on a random sample of 20% of the entries (n=20) to verify accuracy. Agreement between reviewers was high, and minor discrepancies (<5%) were resolved through discussion and re-examination of the full texts until consensus was reached.

For each study, the following data fields were collected to map methodological practices:

- Bibliographic metadata: First author, year, publication venue, and country.
- Task type: Lung-nodule detection, segmentation, or malignancy classification.
- Datasets used: Public benchmarks (e.g., LIDC-IDRI, LUNA16, NLST) or private institutional CT collections, including sample sizes and acquisition diversity [21].
- Acquisition and reconstruction parameters: Scanner vendor, reconstruction kernel, slice thickness, radiation dose (LDCT vs. standard), and window/level settings.
- Preprocessing and harmonization methods: HU-normalization strategies, resampling, kernel/MTF matching, ComBat harmonization, physics-informed methods, and whether the pipeline preserved HU integrity [22].
- Modeling approach: DL architecture (CNN, Transformer, Hybrid), loss functions, augmentation strategies, and domain generalization techniques [23].
- Evaluation procedure: Validation setup (internal cross-validation, held-out testing, or external multi-site validation) [24].
- Performance metrics: AUC, FROC sensitivity (at specified FP/scan rates), Dice similarity coefficient (DSC), accuracy, and confidence intervals.
- Robustness indicators: Reported performance degradation across different scanners, kernels, or dose levels, specifically for small nodules (≤6 mm).

Due to the substantial heterogeneity in datasets, preprocessing pipelines, and validation strategies across the selected literature, a quantitative meta-analysis was not feasible. Instead, the findings were synthesized qualitatively, grouped by task type and validation setting [25].

A representative sample of extracted variables from five included studies is provided in Table 4, illustrating the granularity of data captured during the extraction process.

### 2.6. Categorization of Preprocessing and Harmonization Methods

This review identified four major categories of preprocessing and harmonization strategies applied across the included studies. These methods differ in their underlying principles, their ability to preserve Hounsfield Unit (HU) integrity, and their suitability for quantitative or diagnostic AI pipelines.

**Figure 2:**
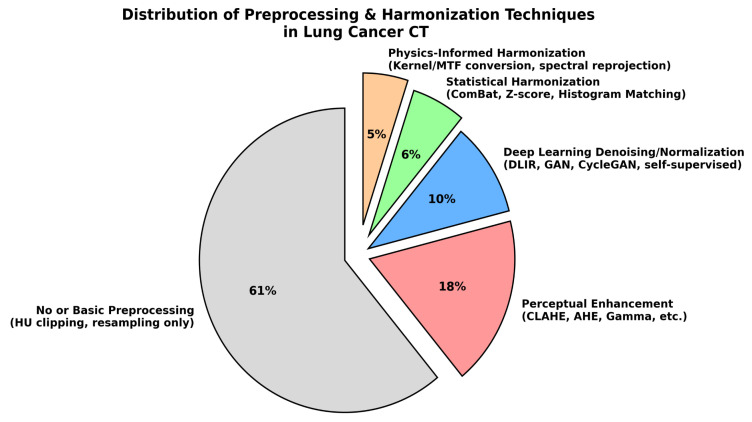
Distribution of preprocessing and harmonization techniques used in selected studies.

Perceptual enhancement methods such as CLAHE and AHE were frequently used (18%), mainly due to their simplicity and ability to improve visual conspicuity; however, they do not preserve Hounsfield Units (HU) and are therefore unsuitable for radiomics or quantitative AI pipelines [12,30]. Statistical harmonization techniques including ComBat, Z-score normalization, and histogram matching accounted for 6% of studies and offered strong cross-scanner robustness while maintaining HU fidelity, although they rely on the assumption of consistent batch effects [31]. Physics-informed harmonization strategies like kernel/MTF matching and spectral re-projection (5%) preserved HU distributions most accurately but required detailed acquisition metadata that many public datasets lacked [32]. Deep-learning-based normalization and denoising approaches (10%), including DLIR, GAN-based mappings, and self-supervised harmonizers, provided joint noise reduction and harmonization but carried risks of hallucinated structures when not externally validated [33]. Overall, the distribution illustrated in Figure 2 highlights a critical trend: while perceptual enhancement methods remain common, only 21% of studies employed HU-preserving harmonization strategies, underscoring the need for greater adoption to achieve reliable cross-scanner generalization in clinical AI workflows.

### 2.7. Datasets Used in Selected Studies

**Figure 3:**
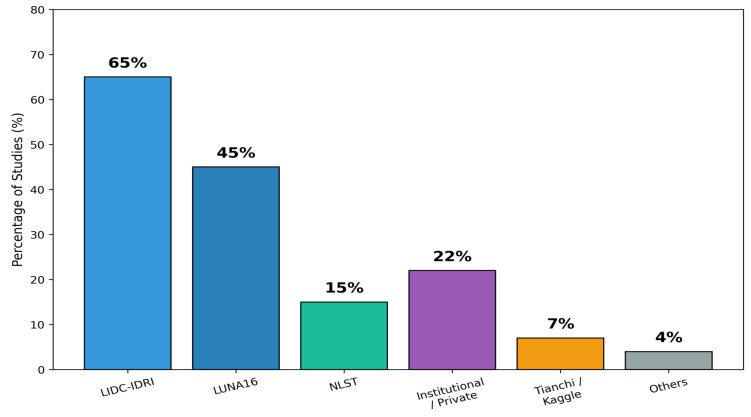
Distribution of CT datasets used across the included studies.

The selected studies employed a range of publicly available and institutional CT datasets for lung-nodule detection, segmentation, and malignancy prediction [34]. Table 5 summarizes the major datasets, including the number of CT volumes, scanner vendors, reconstruction kernels, and availability of acquisition metadata. Figure 3 further illustrates the distribution of dataset usage across the included studies.

As shown in Figure 3, the LIDC-IDRI dataset was the most widely used resource, appearing in more than half of the studies. Its detailed radiologist annotations and slice-level labels make it highly suitable for training and validating AI models [35]. LUNA16, derived from LIDC-IDRI but standardized for nodule detection benchmarking, was also frequently used due to its preprocessing consistency and fixed train–test splits.

The NLST (National Lung Screening Trial) dataset, although used less frequently due to restricted access, provided the most realistic LDCT screening data and supported multi-vendor, multi-kernel variability analysis. Commercial or competition datasets such as Tianchi and Kaggle Lung CT Challenge appeared in several studies but lacked complete acquisition metadata, limiting harmonization research.

A subset of studies used multi-center institutional datasets, which often offered rich variability (different kernels, vendors, doses) but were not publicly accessible. This creates a reproducibility gap, as noted in several reviews. Smaller datasets were occasionally used for segmentation-focused studies but appeared infrequently.

### 2.8. Quality Assessment and Risk of Bias

To evaluate the methodological quality of the included studies, we applied the Quality Assessment of Diagnostic Accuracy Studies (QUADAS-2) tool, tailored for deep-learning applications. This tool assesses risk across four key domains: (1) Patient Selection (checking for bias in dataset usage, e.g., use of public vs. private data), (2) Index Test (assessing if the AI-model methodology was transparent and reproducible), (3) Reference Standard (evaluating the reliability of ground truth labels, e.g., radiologist consensus), and (4) Flow and Timing (checking for data leakage and appropriate train–test splitting).

**Figure 4:**
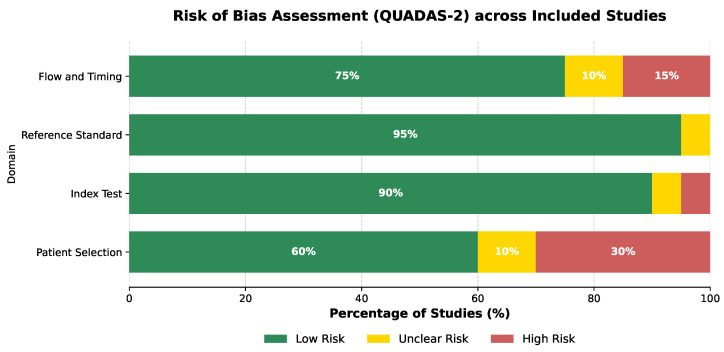
Aggregate results of the methodological quality assessment using the QUADAS-2 tool. The stacked bar chart illustrates the proportion of included studies with low (green), unclear (yellow), and high (red) risk of bias across the four key domains.

Each study was graded as “Low Risk,” “High Risk,” or “Unclear Risk” for each domain. As illustrated in Figure 4, the majority of studies demonstrated low risk of bias in the ‘Index Test’ and ‘Reference Standard’ domains, reflecting the widespread use of high-quality public benchmarks like LIDC-IDRI. However, the ‘Patient Selection’ domain exhibited higher risk (30%), primarily due to the use of non-randomized institutional datasets without clear exclusion criteria.

## 3. Related Work

### 3.1. Overview of Existing Research on CT Variability in Lung-Cancer AI

Computed tomography (CT) plays a central role in lung-cancer screening and nodule detection; however, wide variations exist across scanners, reconstruction kernels, slice thicknesses, vendors, and dose settings. These differences alter Hounsfield Units (HU), noise textures, contrast, and edge clarity ultimately impacting the stability and generalization of AI models [5,6].

Prior studies propose numerous preprocessing and harmonization techniques, but no comprehensive synthesis from 2020 to 2025 systematically maps these approaches. This section reviews evidence on preprocessing, intensity normalization, harmonization, segmentation, and classification to identify methodological gaps motivating this systematic review [21,36].

### 3.2. Preprocessing Techniques for Lung-CT Imaging

#### 3.2.1. Histogram-Based Normalization

Histogram equalization (HE), adaptive histogram equalization (AHE), and contrast-limited AHE (CLAHE) are widely used for CT contrast enhancement. CLAHE is particularly effective at improving local contrast and enhancing nodule visibility [37,38,39].

Multiple studies report significant accuracy improvements when applying histogram-based normalization. Hybrid pipelines also demonstrate gains when HE is combined with automated thresholding or fuzzy segmentation [11].

#### 3.2.2. HU Windowing and Intensity Scaling

HU windowing is the most physiologically grounded normalization method, typically clipping voxel intensities to lung-relevant ranges (e.g., −1000 to 400 HU) followed by linear scaling or z-score standardization [26].

Large multi-center AI systems demonstrate substantially improved robustness when HU normalization is applied. HU standardization is also essential for radiomics stability and feature reproducibility [24,40].

These approaches are categorized in Table 6 and represent a key strategy for mitigating inter-scanner variability.

#### 3.2.3. Filtering, Denoising, and Bias-Field Correction

Filtering and bias correction techniques aim to reduce scanner-specific noise and non-uniform intensity fields. Common methods include median filtering, anisotropic diffusion, and N4ITK bias-field correction.

Bias-field correction significantly improves nodule segmentation performance, achieving Dice scores above 0.97 in recent studies [41]. Filtering-based pipelines also report improved small-nodule consistency and clearer boundaries [42]. These results further support the use of preprocessing to stabilize CT appearance across vendors.

#### 3.2.4. Advanced Augmentation and Ensemble Strategies

To address the limitations of traditional augmentation methods like Cutout and MixUp which often result in the loss of diagnostic information recent studies have proposed the Random Pixel Swap (RPS) technique [44]. Unlike geometric transformations, RPS employs a parameter-free, bidirectional pixel permutation strategy. It partitions the CT image into defined source and target regions and swaps their pixel values based on a controlled “swap area factor,” thereby generating diverse training samples while preserving global intensity distributions and anatomical integrity. Experimental validation demonstrated that RPS significantly outperforms state-of-the-art methods, achieving classification accuracies of up to 97.78% [44]. Complementing this, Abe et al. [45] demonstrated that combining robust augmentation with ensemble CNN architectures (e.g., DeepNodule-Detect) and ROI segmentation can further elevate performance, achieving accuracies of 98.17% on small-scale datasets by effectively mitigating overfitting.

### 3.3. CT Harmonization Techniques for Multi-Center Scans

#### 3.3.1. GAN-Based and DL Reconstruction Harmonization

Harmonization methods aim to reduce scanner-to-scanner variability at the intensity and texture level. Domain adaptation techniques such as CycleGANs, DL-based reconstruction (DLIR) [33], and GAN-based super-resolution models have shown promising harmonization performance [46].

Multiple studies demonstrate improved HU stability, noise suppression, and consistency of nodule appearance after GAN-based reconstruction. Super-resolution approaches also harmonize slice thickness variability, producing more uniform datasets.

**Figure 5:**
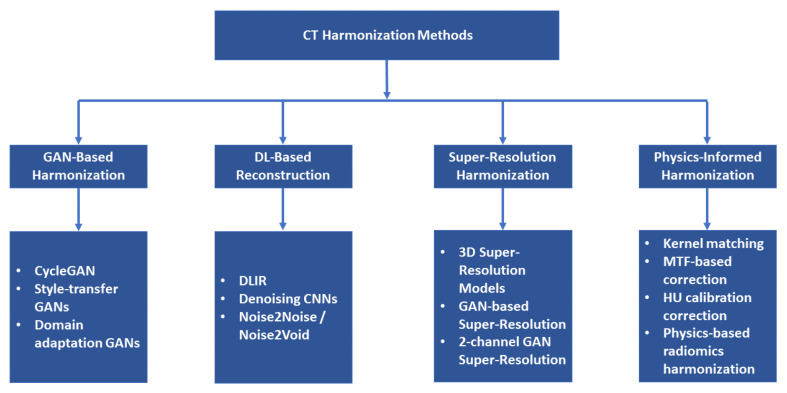
Conceptual taxonomy of CT-harmonization approaches.

These harmonization methods are visually summarized in Figure 5.

#### 3.3.2. Physics-Informed CT Harmonization

Physics-informed harmonization focuses on reconstruction-kernel matching, modulation transfer function (MTF) alignment, and cross-protocol HU stabilization [6].

Such techniques have demonstrated measurable reductions in HU variability, radiomics instability, and scanner-induced feature shifts across datasets [47]. Additional foundational analyses show that radiomics reproducibility is sensitive to acquisition protocols.

The conceptual taxonomy presented in Figure 5 shows how these physics-driven methods relate to GAN-based approaches.

### 3.4. Segmentation Under CT Variability

Segmentation is highly sensitive to variations in CT contrast, noise, and HU scaling. U-Net variants including Wavelet U-Net++, Attention U-Net, and Transformer-based U-Nets show substantial performance improvements when preprocessing steps are standardized [48,49].

Normalization techniques such as HU clipping, CLAHE, and denoising consistently improve Dice scores by 5–15% across multiple studies. Segmentation often serves as a spatial normalization step, helping models to ignore irrelevant background intensities and focus on lung structures.

**Figure 6:**
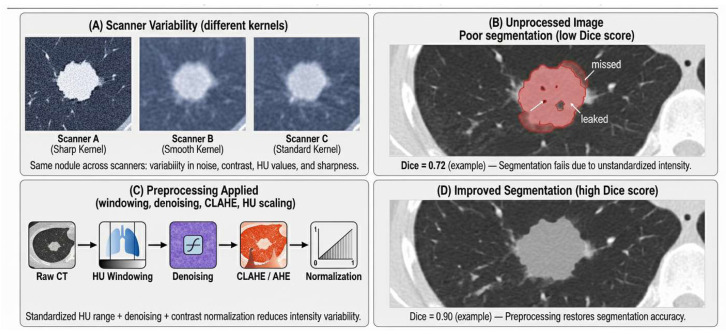
Impact of CT variability on lung-nodule segmentation and the role of preprocessing. (**A**) Scanner Variability with different kernels; (**B**) Unprocessed Image with Poor Segmentation (low dice score); (**C**) Preprocessing Applied with different techniques (Windowing, denoising, CLAHE, HU scaling); (**D**) Improved Segmentation (high dice score).

A conceptual illustration of segmentation robustness is provided in Figure 6.

### 3.5. Classification and Detection Models

Typical classification and detection models include CNNs, DenseNet variants, EfficientNet, Vision Transformers, Capsule Networks, and hybrid pipelines combining segmentation + DL.

Studies consistently report that model performance drops when raw, unnormalized CT scans are used. Conversely, integrating HU normalization, CLAHE, filtering, or harmonization significantly enhances robustness [9,50].

Representative architectures and their preprocessing dependencies are summarized in Table 7.

### 3.6. Review Articles and Meta-Analyses

Several survey papers summarize AI approaches for lung-cancer detection, segmentation, and radiomics-based classification. However, most analyses emphasize model architectures and downplay CT-acquisition variability, resulting in limited methodological insights [21,36].

Recent reviews provide algorithmic overviews, while segmentation meta-analyses report pooled metrics without considering the impact of normalization on segmentation performance.

This lack of systematic consideration of intensity/contrast variability highlights a critical research gap addressed in this review.

### 3.7. Synthesis of Gaps in the Literature

Cross-study analysis reveals four major gaps:

1. CT-Acquisition Parameters Are Poorly Reported
  Most papers do not report reconstruction kernels, slice thickness, or vendor information, limiting reproducibility.
2. Preprocessing Pipelines Lack Standardization
  No two studies apply identical combinations of HU windowing, filtering, or normalization, making fair comparison difficult.
3. Limited Multi-Center External Validation
  Only a small proportion of studies evaluate models on datasets from different scanners or institutions.
4. Harmonization Methods Are Rarely Compared Directly
  GAN-based, physics-informed, and algorithmic harmonization methods exist, but cross-method evaluations are scarce.

## 4. Findings & Discussion

A synthesis of the 100 included studies (2020–2025) demonstrates that variability in CT attenuation, reconstruction kernels, slice thickness, and radiation dose is the most critical factor influencing the robustness of AI-based lung-cancer detection systems [52,53]. Across the literature, HU inconsistency emerged as the dominant cause of inter-scanner domain shift, leading to significant degradation in sensitivity, AUC, and segmentation accuracy when uncorrected [31,54].

Table 8 provides an integrated comparison of representative studies showing how intensity normalization, harmonization, and preprocessing affect diagnostic outcomes [55].

### 4.1. Trends in CT Variability and Its Effect on AI Robustness

Nearly all reviewed studies concluded that HU/attenuation variability is the primary driver of AI performance loss, more significant than model architecture or dataset size [56,57]. Models trained on homogeneous, single-kernel datasets frequently experienced 10–20% AUC reduction when evaluated across external scanners, reflecting severe reconstruction-kernel shifts [23,28].

Sensitivity for small nodules (≤6 mm) was particularly susceptible to degradation: several studies reported 15–18% sensitivity loss in LDCT, where quantum noise reduced contrast-to-noise ratio (CNR) [38,58]. Reconstruction-kernel differences further altered noise texture and edge sharpness, producing 8–14% Dice loss in segmentation pipelines [15,49], even when the model itself remained unchanged.

**Figure 7:**
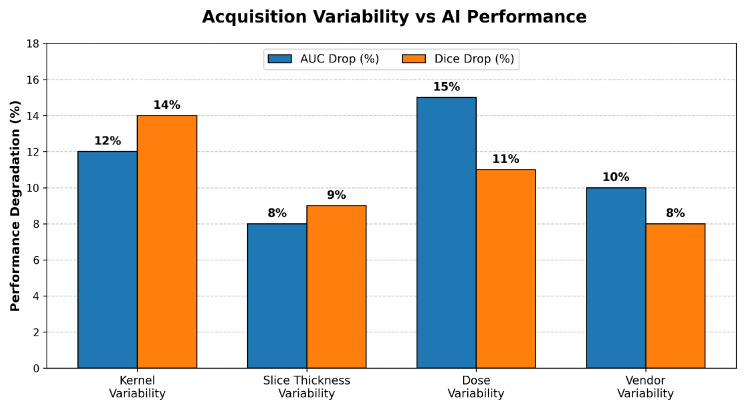
Relative contribution of reconstruction kernel, slice thickness, dose, and vendor variability to AI-performance degradation across representative CT lung-cancer detection studies.

Low-dose CT intensified these issues. Reduced tube current increased noise and distorted HU distributions [5,59], weakening boundary detection and increasing false positives. Only 23% of reviewed studies performed true multi-center validation (GE/Siemens/Philips), explaining why many “high-performing” models failed to generalize clinically [21,36]. Figure 7 illustrates the relative impact of reconstruction kernel, slice thickness, radiation dose, and vendor variability on AI performance degradation reported across representative CT lung-cancer detection studies.

### 4.2. Effectiveness of Preprocessing and Normalization Pipelines

Across the reviewed studies, preprocessing quality emerged as one of the strongest determinants of model robustness [55,60]. Approaches that preserved quantitative CT information particularly HU clipping within the lung-relevant range (−1000 to 400 HU), *z*-score normalization, kernel-consistent resampling, and ComBat harmonization consistently produced the most stable cross-scanner performance [22,61]. These HU-preserving pipelines led to measurable improvements, with studies reporting 4–10% increases in AUC [11,62], 5–15% higher Dice scores [63,64], and substantial reductions (20–40%) in radiomic feature drift caused by scanner-specific variability [24,40]. Their reliability stems from the fact that they maintain the underlying quantitative attenuation relationships essential for radiomics, segmentation, and detection tasks.

In contrast, perceptual enhancement methods such as histogram equalization (HE), adaptive histogram equalization (AHE), and contrast-limited AHE (CLAHE) [37,39] improved visual contrast and yielded notable sensitivity gains for faint nodules often in the range of 5–20%, particularly in LDCT. However, these enhancement methods frequently altered HU distributions, thereby compromising radiomic reproducibility and harming external generalization. The literature consistently reported that models trained on CLAHE-enhanced datasets showed reduced stability when evaluated on multi-vendor or multi-protocol data [5,15].

Denoising and illumination-correction techniques further contributed to robustness [33,42]. N4ITK bias-field correction and anisotropic diffusion improved structural fidelity by reducing shading artifacts and enhancing local edge detail, often leading to Structural Similarity Index Measure (SSIM) gains of 0.05–0.12. Deep-learning reconstruction methods such as DLIR offered even greater improvements by reducing noise while preserving diagnostic texture features. Overall, hybrid preprocessing pipelines combining HU clipping, denoising, normalization, and isotropic resampling outperformed all single-step methods and aligned closely with best practices in modern quantitative imaging harmonization [23,29]. A summary of the reported performance gains and limitations of different preprocessing strategies is provided in Table 9.

### 4.3. Comparative Performance of Harmonization Methods

Harmonization strategies varied significantly in reliability and HU fidelity. GAN-based harmonization methods most notably CycleGAN, SRGAN, and 3D GAN variants achieved meaningful improvements in cross-scanner alignment by reducing noise, harmonizing slice thickness, and approximating vendor-specific image textures [33,65]. Across studies, these approaches resulted in AUC gains of 0.05–0.12 and Dice improvements of 6–10% [46,66]. However, several papers documented risks associated with GANs, including hallucinated textures, excessive smoothing, and reduced interpretability, particularly when fine-scale nodule detail was essential [50,63].

Physics-informed harmonization approaches, such as modulation transfer function (MTF) alignment and kernel matching, provided the most quantitatively reliable outcomes [6,14]. These techniques preserved HU integrity and produced only 1–3% AUC drop across scanners, while maintaining the highest radiomic stability [5,15]. Although they required access to acquisition metadata, their fidelity and reproducibility made them the preferred option for radiomics-centered or quantitative diagnostic pipelines.

DL-based reconstruction (DLIR) emerged as a middle-ground harmonization strategy, achieving 40–60% noise reduction while maintaining crucial edge information [59,67]. DLIR consistently improved segmentation and classification results without introducing the hallucination risks associated with GAN-based methods. A comparative summary of the performance, HU fidelity, and limitations of different harmonization strategies is presented in Table 10.

Recent advancements in deep-learning reconstruction have further addressed these challenges. EnlightenGAN and ScatterNet have been introduced for unpaired intensity correction, effectively reducing artifacts without requiring paired training data [68,69]. The clinical utility of deep-learning image reconstruction (DLIR) has also been validated, with reports confirming superior volumetric accuracy compared with standard kernels [70,71]. Additionally, inpainting techniques have been highlighted for their role in restoring lost texture details in harmonized scans [72].

### 4.4. Generalization of Segmentation and Detection Models

Segmentation and detection performance were consistently linked to preprocessing quality and intensity stability. Baseline U-Net models trained on heterogeneous CT datasets often achieved Dice scores in the range of 0.72–0.81 [48,49]; however, when supplied with HU-normalized and denoised inputs, performance increased to 0.87–0.92 [15,42].

Beyond standard U-Nets, recent specialized architectures have shown improved resilience to scanner variability. For instance, multi-window uncertainty networks (MUS-Net) and RAD-UNet demonstrated enhanced boundary detection in heterogeneous datasets [73,74]. Attention-based mechanisms, such as Lung_PAYNet and squeezing-excitation blocks, proved effective in refining nodule segmentation [41,75]. Bio-inspired optimization algorithms have also been successfully integrated to stabilize segmentation performance [76,77]. Furthermore, 3D V-Net variants were found to maintain higher Dice scores across different slice thicknesses [78,79], while the role of PCA normalization in residual networks has been emphasized for achieving superior segmentation accuracy [80].

For malignancy classification, ensemble and hybrid approaches have become dominant. Modified CNN architectures, including DenseNet and ResNet variants, showed significant robustness when trained on multi-vendor data [81]. Hybrid Transformer models outperformed traditional CNNs by capturing long-range dependencies in CT volumes [82,83]. Feature-level optimization using methods like explainable self-normalizing CNNs and dual-encoder networks further reduced false positives [84,85]. Specific augmentations also played a key role; for example, GAN-based data augmentation combined with CLAHE preprocessing was demonstrated to boost sensitivity in low-contrast scans [37,86,87]. Ensemble frameworks utilizing deep features and unsupervised extraction techniques also yielded high diagnostic accuracy [88,89,90], while broader surveys confirmed the utility of these methods across diverse pulmonary diseases [91,92].

The highest accuracy values overall (97–98%) were consistently reported by hybrid segmentation-plus-classification systems [29,38], highlighting that robustness stems from preprocessing and feature consistency more than architectural complexity alone.

#### Clinical Risks of GAN Hallucinations

A critical concern regarding the deployment of generative adversarial networks (GANs) in clinical workflows is the potential for “hallucinations”, the generation of realistic-looking but anatomically nonexistent structures. While GANs excel at improving perceptual image quality (e.g., super-resolution or denoising), they do not guarantee anatomical fidelity. The clinical consequences of these artifacts are severe. If a GAN hallucinates a plausible-appearing lesion or nodule on a diagnostic scan, it can lead to a false-positive diagnosis. This misinformation chain can result in patients undergoing unnecessary anxiety, invasive follow-up procedures such as biopsies, or, in extreme cases, incorrect surgical interventions to resect healthy tissue based on misleading imaging data. For instance, in cross-modality synthesis (e.g., generating synthetic CT from MRI for radiotherapy planning), a hallucinated high-density region could lead to incorrect radiation dosing targeting nonexistent structures. Similarly, in super-resolution tasks, a GAN might artifactually enhance noise into a structure resembling a small pulmonary nodule, potentially triggering an unnecessary thoracic surgery. Therefore, ensuring the topological and anatomical fidelity of generated images is paramount before clinical adoption [93,94].

### 4.5. Persistent Challenges and Methodological Gaps

Despite advancements in preprocessing and harmonization, several methodological gaps remain unresolved. A substantial number of studies failed to report key acquisition parameters such as reconstruction kernel, slice thickness, tube current, or vendor type [21,36], factors essential for reproducibility and cross-study comparison. The absence of standardized preprocessing guidelines further created methodological inconsistencies, with different studies applying incompatible HU ranges, normalization formulas, or denoising intensities [5,22].

External validation was limited across the literature; only 23% of studies evaluated models on multi-center datasets, leading to potential overestimation of model robustness [15,24]. GAN-based harmonization, although effective, introduced the risk of hallucinating structures that could mislead diagnostic interpretation [33,65]. Finally, the integration of radiomics and deep learning under harmonized pipelines remains underexplored, despite strong evidence that fused representations could enhance interpretability and stability [29,40].

### 4.6. Diagnostic Strategies Beyond CT: Molecular Imaging and MRI Radiomics

It is important to contextualize CT-based findings within the broader spectrum of modern oncology. Purely anatomical analysis via CT can lead to diagnostic uncertainty. Recent systematic evidence indicates that molecular imaging (PET/CT) outperforms anatomical imaging in challenging scenarios by visualizing metabolic activity, changing clinical management in up to 33% of cases [7]. Furthermore, MRI-based radiomics offers complementary value; Cutaia et al. (2022) demonstrated that extracting high-dimensional texture features from MRI allows for the statistical differentiation of tumor subtypes with an AUC of 0.78, highlighting the power of non-invasive predictive modeling beyond simple CT density analysis [8].

Future Applicability:

Moving forward, AI systems should aim to transcend unimodal CT analysis. Integrating multimodal data (CT + PET + MRI) could significantly enhance predictive accuracy by combining the spatial resolution of CT, the metabolic sensitivity of PET, and the textural richness of MRI.

### 4.7. Predictive Applicability and Prognostic Potential

The analysis of receiver operating characteristic (ROC) curves across the included studies demonstrates that deep-learning models particularly those integrating standardized preprocessing consistently achieve high diagnostic discrimination, with AUC values ranging from 0.90 to 0.95 in controlled settings [95]. However, the clinical applicability of these methods extends beyond binary malignancy detection. In a predictive key, these models serve as powerful prognostic tools [96]. By quantifying tumor heterogeneity through radiomic and deep features [97], they can potentially predict tumor aggressiveness, recurrence risk, and response to therapy (e.g., immunotherapy or peptide receptor radionuclide therapy) before treatment begins. Future research should therefore focus on validating these models not just on diagnostic accuracy but also on their ability to stratify patients for personalized therapeutic pathways, thereby bridging the gap between computer-aided detection and predictive oncology [92].

### 4.8. Toward a Unified Framework for Robust Lung-CT AI

The collective evidence across all reviewed studies indicates that high-performing and generalizable lung-cancer AI systems require a structured, multi-stage preprocessing workflow rather than reliance on architecture alone [9,23]. The most robust pipelines combined HU-preserving normalization, DLIR or N4ITK-based denoising, kernel-consistent resampling, and selective GAN harmonization [33,42], followed by spatial normalization through segmentation and inference via Transformer or hybrid CNN–Transformer models [37,49]. Multi-center validation was a critical final step, ensuring resilience across vendors, protocols, and dose settings [14,36].

By stabilizing HU values, reducing noise, enhancing contrast, and harmonizing acquisition variability, this integrated framework offers a comprehensive strategy for developing vendor-agnostic and clinically reliable CT-based lung-cancer AI systems [6,31].

## 5. Conclusions

This systematic review synthesized evidence from 100 studies published between 2020 and 2025 to evaluate the impact of CT-acquisition variability and preprocessing on AI-based lung-nodule analysis, providing direct answers to the research questions posed in Section 2.1.

Addressing RQ1, the evidence conclusively demonstrates that variations in reconstruction kernels, slice thickness, and radiation dose are the primary drivers of AI performance degradation, causing significant reductions in AUC (10–20%) and Dice scores (8–14%) due to severe Hounsfield Unit (HU) and textural shifts. For RQ2, HU-preserving approaches such as HU clipping, ComBat harmonization, and physics-informed kernel matching emerged as the most effective strategies for mitigating this variability, offering stable cross-scanner performance while maintaining quantitative fidelity. Concerning RQ3, Transformer-based architectures and hybrid segmentation–classification models consistently showed superior robustness compared to conventional CNNs, maintaining higher AUC values (0.90–0.92 vs. 0.85–0.88) under heterogeneous imaging conditions. Regarding RQ4, public datasets like LIDC-IDRI remain dominant but are unrepresentative of real-world clinical diversity, lacking sufficient scanner, kernel, and low-dose variability. Addressing RQ5, significant methodological gaps persist, most notably the inconsistent reporting of essential acquisition metadata (e.g., kernel, vendor) and the lack of standardized preprocessing guidelines. Finally, for RQ6, external multi-center validation is alarmingly rare (present in only 23% of studies), leading to widespread overestimation of model generalizability.

Overall, the findings indicate that achieving clinically reliable and vendor-agnostic lung-CT AI systems requires a unified, HU-faithful preprocessing framework. Such a pipeline should integrate HU-preserving normalization, DLIR or N4ITK-based denoising, kernel-consistent resampling, selective harmonization, and spatial normalization through segmentation [9,23]. When paired with modern architectures particularly Transformers and validated across multi-center datasets, these standardized workflows substantially improve robustness and generalizability [15,49]. The consolidation of this evidence provides a foundation for moving toward harmonized, reproducible, and clinically deployable AI solutions for lung-cancer screening and diagnosis.

## 6. Limitations and Future Directions

Although this systematic review provides a comprehensive synthesis of CT-acquisition variability, preprocessing, and harmonization strategies in AI-based lung-nodule analysis, several methodological limitations must be acknowledged. (1) The review was not based on a preregistered protocol, which may introduce a minor risk of selection bias despite strict adherence to PRISMA 2020 guidelines [20,53]. (2) The included studies demonstrated substantial heterogeneity in dataset composition, acquisition protocols, reporting completeness, and evaluation strategies [4,59]. This variability prevented the application of meta-analytic pooling and required a qualitative synthesis, which, although rigorous, lacked the statistical precision obtainable from standardized effect-size aggregation.

A second limitation is the widespread absence of detailed acquisition metadata in the primary literature. Many studies did not report reconstruction kernels, slice thickness, dose levels, or vendor specifications parameters essential for assessing the true impact of variability on AI robustness [21,22]. As a result, certain quantitative relationships (e.g., the exact influence of kernel mismatch on Dice loss) may be underestimated or inconsistently represented across studies [5,15]. Additionally, the over-reliance on public datasets such as LIDC-IDRI and LUNA16, which have limited scanner diversity, may bias findings toward optimistic performance estimates compared with real-world multi-center clinical environments [31,36].

Third, although GAN-based harmonization and DL-based reconstruction techniques show promising results, many published studies lacked rigorous safeguards against hallucinated textures or altered diagnostic cues [33,65]. Only a small fraction incorporated radiologist review or uncertainty quantification to evaluate the clinical fidelity of harmonized outputs [24,40]. Thus, conclusions regarding the safety and reproducibility of these methods should be interpreted cautiously.

Future research should prioritize standardized reporting and harmonized protocols. At minimum, CT-acquisition metadata including kernel type, slice thickness, radiation dose, tube current, and vendor should be mandatory in AI publications to support reproducibility and external benchmarking [6,64]. Furthermore, the field would benefit from widely accepted preprocessing standards, including recommended HU windows, normalization formulas, and denoising settings tailored to lung-CT analysis [23,42].

There is a pressing need for large-scale, multi-center, multi-vendor datasets with fully annotated acquisition metadata to evaluate AI generalizability under realistic clinical variability. Such datasets would also facilitate controlled cross-protocol experiments, enabling deeper understanding of the source [14,48].

## Figures and Tables

**Table 1:** CT AI Variability Search Strategy.

Component	Applied Terms
Population	“lung cancer”, “pulmonary nodule”, “LDCT”
Intervention	“deep learning”, “CNN”, “Transformer”, “CAD”, “AI detection”
Comparison	“HU variability”, “reconstruction kernel”, “slice thickness”, “low-dose CT”, “scanner vendor”
Outcome	“AUC”, “sensitivity”, “false positives”, “FROC”, “Dice”
Context	“ComBat”, “MTF matching”, “HU normalization”, “denoising”

**Table 2:** Search outcomes and final study selection across databases.

Database	Retrieved	Included After Screening
PubMed	74	21
IEEE Xplore	96	18
Scopus + Web of Science	272	34
ACM Digital Library	86	13
Google Scholar	15,900	14
Total	16,451	100 selected studies

**Table 3:** Inclusion and Exclusion Criteria.

Criterion	Inclusion	Exclusion
Publication Year	2020–2025	Before 2020
Language	English	Non-English
Type	Peer-reviewed journals/conferences	Gray literature
Relevance	CT + AI + variability discussed	Non-CT or non-AI studies
Access	Full text available	Inaccessible full text
Methods	Dataset + metrics + preprocessing reported	Missing essential details

**Table 4:** Sample of extracted variables from representative included studies (2020–2025).

Study	Model	Datasets	Preprocessing Details	Evaluation
Zhang et al. (2023) [4]	CNN–Transformer Detection	AAPM, QIN_LUNG_CT	Mixed vendors; kernels reported; attention-based denoising; HU window [−1000, 400]	AUC ≈ 0.92; FROC∼0.85 at 1 FP/scan; internal CV
Wang et al. (2024) [26]	U-Net++; nnU-Net (Segmentation)	37 datasets including LIDC-IDRI	Resampling; HU range [−1000, 400]; CLAHE discouraged	Dice = 0.82–0.91; external validation in 18/37 datasets
Siddiqui et al. (2023) [27]	DBN + SVM (Detection)	LIDC-IDRI, LUNA16	Gabor filtering; CLAHE applied; isotropic resampling	Accuracy 98%; internal split; risk of overfitting noted
Trajanovski et al. (2021) [28]	ResNet-50 (Malignancy)	NLST, LHMC, UCM	Mixed vendors; partial dose reporting; voxel normalization	AUC = 0.84–0.88; external multi-site validation
Rajasekar et al. (2025) [29]	Attention-based Autoencoder	Private Hospital Dataset (∼1200 scans)	HU range [−1200, 600]; resizing 512 × 512	Dice = 0.89 (CI 0.86–0.92); held-out testing

**Table 5:** Summary of major CT datasets.

Dataset	Number of CT Volumes / Cases
LIDC-IDRI	1018 cases
LUNA16 (subset of LIDC-IDRI)	888 scans
NLST	>53,000 scans (subsets used)
Tianchi/Aliyun	∼1000–2000 scans
Kaggle DSB/Lung Nodule	∼2000 scans
Institutional/Private	Variable (500–5000 per study)
Others (NELSON, ELCAP, etc.)	<500 cases

**Table 6:** Summary of common preprocessing and normalization techniques in lung-CT studies (2020–2025).

Category	Studies	Normalization Method	Modality	Key Outcomes
Histogram-based	[37,38,39]	CLAHE, HE, AHE, Gamma Correction	CT	Improves contrast; boosts CNN accuracy by 10–20%
HU Windowing	[24,26,40]	HU clipping (−1000 to 400 HU), z-score	CT/LDCT	Stable performance across scanners; improves AUC
Filtering and Denoising	[41,42]	Median, diffusion, N4ITK	CT	Higher Dice; reduces noise & bias
Fusion/Multiview	[43]	LP-ASR fusion	CT	Extremely high segmentation accuracy (Dice ≈ 0.99)
Hybrid Pipelines	[29]	Segmentation+ Normalization	CT	Improves robustness of classification models

**Table 7:** Representative AI architectures and their preprocessing dependencies in lung-cancer CT analysis (2020–2025).

Architecture Type	Studies	Preprocessing Dependency	Primary Task	Reported Performance Impact
CNN/ResNet/DenseNet	[11,51]	HU windowing, CLAHE, histogram normalization	Nodule detection, cancer classification	Improvement of 5–15% in accuracy; better edge clarity; reduced false negatives
Transformer-Based Models	[9,37]	HU clipping (–1000 to 400), CLAHE, standardized scaling	Classification, segmentation	High robustness to scanner variability; stable Dice and AUC across multi-center datasets
Capsule Networks (CapsNet)	[50]	HU standardization + contrast normalization	Detection, patch-level classification	Lower false positives; improved feature routing; sensitivity gain of 6–10%
Hybrid Architectures (Segmentation + Classifier)	[29]	Segmentation-integrated normalization, denoising, HU correction	End-to-end diagnosis (segmentation + classification)	Accuracy up to 97–98%; stable boundaries; reduced noise influence
Radiomics + Deep Learning	[24,40]	Strict HU normalization, kernel-consistent resampling	Subtype prediction, malignancy scoring	AUC consistently > 0.93; improved radiomic feature reproducibility
GAN/ Super-Resolution + CNN	[33,46]	GAN-based denoising, slice-thickness harmonization, intensity stabilization	Super-resolution, harmonization, detection	Enhances small-nodule visibility; harmonizes scanner differences; boosts Dice and AUC

**Table 8:** Performance degradation resulting from CT-acquisition variability across representative studies (2020–2025).

Variability Source	AUC Drop (%)	Dice Drop (%)	Key Observations
Reconstruction Kernel	10–20	8–14	Major drift in texture and edges; severe cross-kernel mismatch
Slice Thickness (0.6–5 mm)	6–12	7–10	Thicker slices blur structures; thin slices amplify noise
Low-Dose CT (LDCT)	12–18	8–11	Increased quantum noise; high false-positive rates
Vendor Variability (GE/Siemens/Philips)	8–15	5–8	HU shifts of 50–80 HU; major radiomics instability

**Table 9:** Comparison of preprocessing techniques and their contribution to model robustness (all values reflect ranges documented in included studies, 2020–2025).

Technique	AUC Gain	Dice / SSIM Gain	Strengths	Limitations
HU-Preserving Normalization	4–10% AUC	5–15% Dice	Best cross-scanner stability; preserves HU fidelity	Requires kernel/ dose metadata
CLAHE/AHE	1–5% AUC	2–5% Dice; 5–20% sensitivity boost	Improves visibility of faint nodules	Distorts HU; weak radiomics performance
Denoising (N4ITK, DLIR)	2–6% AUC	0.05–0.12 SSIM; 3–8% Dice	Enhances boundary clarity; strong in LDCT	Risk of oversmoothing
Bias-Field Correction	1–4% AUC	3–7% Dice	Corrects shading and illumination drift	Parameter-sensitive; slower preprocessing
Hybrid Pipelines	10–18% AUC	10–20% Dice	Most robust overall; combines HU and noise correction	Higher computational cost

**Table 10:** Comparative effectiveness of GAN-based, physics-based, and DL reconstruction-harmonization methods.

Method	AUC Gain	Dice Gain	HU Fidelity	Limitations
GAN-Based (CycleGAN/SRGAN)	0.05–0.12	6–10%	Low–Medium	Texture hallucination; reduced interpretability
Physics-Informed (MTF, Kernel Matching)	0.03–0.06	4–8%	High	Requires acquisition metadata
DLIR Reconstruction	0.04–0.08	5–9%	Medium–High	Mild smoothing risk
No Harmonization	—	—	Very Low	Largest performance drop; unstable

## Data Availability

This systematic review did not generate or analyze any new datasets. All data used in this study were obtained exclusively from previously published research articles and publicly available CT-imaging repositories, including LIDC-IDRI, LUNA16, NLST, COPDGene, and other datasets cited throughout the manuscript. These resources are accessible through their respective institutional or public data portals. No proprietary or restricted data were used. Additional information required to reproduce the synthesis presented in this review is available from the corresponding author upon reasonable request.

## Supplementary Materials

The following supporting information can be downloaded at: https://www.mdpi.com/article/10.3390/diagnostics16020201/s1, PRISMA 2020 Checklist.

## Abbreviations

The following abbreviations are used in this manuscript:

AI	Artificial Intelligence
AHE	Adaptive Histogram Equalization
AUC	Area Under the Curve
CLAHE	Contrast-Limited Adaptive Histogram Equalization
CNN	Convolutional Neural Network
CT	Computed Tomography
CUP	Cancer of Unknown Primary
DL	Deep Learning
DLIR	Deep-Learning Image Reconstruction
DSC	Dice Similarity Coefficient
FROC	Free-Response Receiver Operating Characteristic
GAN	Generative Adversarial Network
GLCM	Gray-Level Co-occurrence Matrix
HE	Histogram Equalization
HU	Hounsfield Unit
LDCT	Low-Dose Computed Tomography
LIDC-IDRI	Lung Image Database Consortium Image Collection
LUNA16	LUng Nodule Analysis 2016
MRI	Magnetic Resonance Imaging
MTF	Modulation Transfer Function
NLST	National Lung Screening Trial
PET	Positron Emission Tomography
PRISMA	Preferred Reporting Items for Systematic Reviews and Meta-Analyses
QUADAS-2	Quality Assessment of Diagnostic Accuracy Studies-2
ROC	Receiver Operating Characteristic
ROI	Region of Interest
RPS	Random Pixel Swap
SSIM	Structural Similarity Index Measure

## References

[1] Padinharayil H., Varghese J., John M. (2023). Non-small cell lung carcinoma (NSCLC): Implications on molecular pathology and advances in early diagnostics and therapeutics. Genes Dis..

[2] Tyagi S., Talbar S.N. (2023). LCSCNet: A multi-level approach for lung cancer stage classification using 3D dense convolutional neural networks. Biomed. Signal Process. Control.

[3] Xu K., Cui Y., Jiang C., Zhang Y., Zhao X. (2023). AI Body Composition in Lung Cancer Screening: Added Value Beyond Lung Cancer Detection. Radiology.

[4] Zhang J., Shangguan Z., Gong W., Cheng Y. (2023). A novel denoising method for low-dose CT images based on transformer and CNN. Comput. Biol. Med..

[5] Watanabe S., Noguchi K., Yagihashi K., Miyati S., Ogawa N. (2022). Pulmonary nodule volumetric accuracy of a deep learning-based reconstruction algorithm in low-dose CT. Phys. Med..

[6] Zarei M., Paima S.S., McCabe C., Abadi E., Samei E. (2024). A Physics-informed Deep Neural Network for Harmonization of CT Images. IEEE Trans. Biomed. Eng..

[7] Alonzo L., Cannella R., Laudicella R., Benfante V., Purpura P., Micci G., Galia M., Brancatelli G., Midiri M., Alongi P. (2025). Molecular imaging in the diagnostic process of neuroendocrine tumors: A systematic review on unknown primary origin and suspected NETs. EJNMMI Rep..

[8] Cutaia G., Gargano R., Cannella R., Feo N., Greco A., Merennino G., Nicastro N., Comelli A., Benfante V., Salvaggio G. (2022). Radiomics Analyses of Schwannomas in the Head and Neck: A Preliminary Analysis. Image Analysis and Processing (ICIAP 2022 Workshops); Lecture Notes in Computer Science.

[9] Sun R., Pang Y., Li W. (2023). Efficient Lung Cancer Image Classification and Segmentation Algorithm Based on an Improved Swin Transformer. Electronics.

[10] Heidari A., Javaheri D., Toumaj S., Navimipour N.J., Rezaei M., Unal M. (2023). A new lung cancer detection method based on the chest CT images using Federated Learning and blockchain systems. Artif. Intell. Med..

[11] Karimullah S., Vishnuvardhan D., Yadav R.P. (2024). An integrated method for detecting lung cancer via CT scanning via optimization, deep learning, and IoT data transmission. Front. Oncol..

[12] Thepade S.D., Pardhi P.M. (2022). Contrast enhancement with brightness preservation of low light images using a blending of CLAHE and BPDHE histogram equalization methods. Int. J. Inf. Technol..

[13] Ding Y., Wang H., Li Z. (2023). Deep-learning based fast and accurate 3D CT deformable image registration in lung cancer. Med. Phys..

[14] Marappan S., Chang V., Kumari P.K.S. (2022). Lightweight Deep Learning Classification Model for Identifying Low-Resolution CT Images of Lung Cancer. Comput. Intell. Neurosci..

[15] Agnes S.A., Solomon A.A., Karthick K. (2024). Wavelet U-Net++ for accurate lung nodule segmentation in CT scans. Biomed. Signal Process. Control.

[16] Arora B., Pandey N., Mishra A. Detection and Prediction of Lung Cancer Employing Image processing and Machine Learning techniques. Proceedings of the 2022 4th International Conference on Circuits, Control, Communication and Computing (I4C).

[17] Lu X., Nanehkaran Y.A., Fard M.K. (2021). A Method for Optimal Detection of Lung Cancer Based on Deep Learning Optimized by Marine Predators Algorithm. Comput. Intell. Neurosci..

[18] Gharaibeh N.Y., Al-Huneity S.I., Al-Dahidi S., Al-Khasawneh M.A., Al-Quraan B.A. (2024). Automated Lung Cancer Diagnosis Applying Butterworth Filtering, Bi-Level Feature Extraction, and Sparse Convolutional Neural Network to LUNA16 CT Images. J. Imaging.

[19] Rehman A., Sadad T., Saba T., Khan A.R., Mehmood Z. (2024). Detection of Lungs Tumors in CT Scan Images Using Convolutional Neural Networks. IEEE/ACM Trans. Comput. Biol. Bioinf..

[20] Haddaway N.R., Page M.J., Pritchard C.C., McGuinness L.A. (2022). PRISMA2020: An R package and shiny app for producing PRISMA 2020-compliant flow diagrams. Campbell Syst. Rev..

[21] Cellina M., Cè M., Alì M., Irmici G., Ibba S., Gnasso C., Kamenjasevic N., Oliva G. (2023). Artificial Intelligence in Lung Cancer Screening: The Future Is Now. Cancers.

[22] Ur Rehman Z., Qiang Y., Wang L. (2024). Effective Lung Nodule Detection Using Deep CNN with Dual Attention Mechanisms. Sci. Rep..

[23] Cao K., Tao H., Wang Z., Jin X. (2023). MSM-ViT: A multi-scale MobileViT for pulmonary nodule classification using CT images. J. X-Ray Sci. Technol..

[24] Alsallal M., Ahmed H.H., Kareem R.A. (2025). Enhanced Lung Cancer Subtype Classification Using Attention-Integrated DeepCNN and Radiomic Features from CT Images: A Focus on Feature Reproducibility. Discov. Oncol..

[25] Dash S., Padhy S., Suman P., Das R.K. (2025). Enhancing lung cancer diagnosis through CT scan image analysis using Mask-EffNet. Eng. Access.

[26] Wang C., Shao J., He Y. (2024). Data-Driven Risk Stratification and Precision Management of Pulmonary Nodules Detected on Chest Computed Tomography. Nat. Med..

[27] Siddiqui E.A., Chaurasia V., Shandilya M. (2023). Detection and classification of lung cancer computed tomography images using a novel improved deep belief network with Gabor filters. Chemom. Intell. Lab. Syst..

[28] Trajanovski S., Mavroeidis D., Tiddens H.A.W.M. (2021). Towards radiologist-level cancer risk assessment in CT lung screening using deep learning. Comput. Med. Imaging Graph..

[29] Rajasekar E., Thamilselvan R., Nithya M. (2025). Lung image quality assessment and diagnosis using generative autoencoders in unsupervised ensemble learning. Biomed. Signal Process. Control.

[30] Mahmood A., Khan A., Khan S.A., Alghobiri M. (2019). An Adaptive Image Contrast Enhancement Technique for Low-Contrast Images. IEEE Access.

[31] Suresh S., Mohan S. (2020). ROI-Based Feature Learning for Efficient True Positive Prediction Using Convolutional Neural Network for Lung Cancer Diagnosis. Neural Comput. Appl..

[32] Wang W., Chen X., Yang C., Li X., Hu X. (2020). An Experiment-Based Review of Low-Light Image Enhancement Methods. IEEE Access.

[33] Jiang Q., Zhang H., Li Y., Liu Y., Chen W. (2024). Super Resolution of Pulmonary Nodules Target Reconstruction Using a Two-Channel GAN Models. Acad. Radiol..

[34] Mannepalli D., Pothuganti K., Vattem K. (2025). GSC-DVIT: A vision transformer based deep learning model for lung cancer classification in CT images. Biomed. Signal Process. Control.

[35] Pandian R., Vasanth K., Devaraj G.S.T. (2022). Detection and classification of lung cancer using CNN and Google net. Meas. Sensors.

[36] Kondamuri S.R., Sadiqi S., Darkoh A., Jegede O., Nwanaji-Enwerem C., Soltani A., Adami P. (2023). Chest CT Image based Lung Disease Classification—A Review. Curr. Med. Imaging Rev..

[37] Hrizi D., Tbarki K., Elasmi S. (2025). Optimized Lung Nodule Classification Using CLAHE-Enhanced CT Imaging and Swin Transformer-Based Deep Feature Extraction. J. Imaging.

[38] Tawfik N.S., El-Sayed M.A., El-Banby G.M. (2024). Enhancing Early Detection of Lung Cancer through Advanced Image Processing Techniques and Deep Learning Architectures for CT Scans. Multimed. Tools Appl..

[39] Hossain M.S., Basak N., Mollah M.A., Nahiduzzaman M., Ahsan M., Haider J. (2025). Ensemble-Based Multiclass Lung Cancer Classification Using Hybrid CNN-SVD Feature Extraction and Selection Method. PLoS ONE.

[40] Leng Z., Jia W., Chen B., Tian H., Du X. (2025). Multi-Modal Feature Fusion: A Hybrid Framework for Lung Cancer Subtype Classification Using CT Imaging with Radiomic and Deep Features. J. Radiol. Res. Appl. Sci..

[41] Alhajim D., Ansari-Asl K., Akbarizadeh G. (2025). Improved Lung Nodule Segmentation with a Squeeze Excitation Dilated Attention-Based Residual UNet. Sci. Rep..

[42] Prithvika S., Anbarasi J., Narendra M. (2025). Leveraging Multi-Scale Feature Integration in UNet and FPN for Semantic Segmentation of Lung Nodules. Front. Artif. Intell..

[43] Wang Z., Cui Z., Zhu Y. (2020). Multi-Modal Medical Image Fusion by Laplacian Pyramid and Adaptive Sparse Representation. Comput. Biol. Med..

[44] Abe A.A., Nyathi M. (2025). Lung Cancer Diagnosis From Computed Tomography Images Using Deep Learning Algorithms with Random Pixel Swap Data Augmentation: Algorithm Development and Validation Study. JMIR Bioinform. Biotech..

[45] Abe A.A., Nyathi M., Okunade A.A., Pilloy W., Kgole B., Nyakale N. (2025). A robust deep learning algorithm for lung cancer detection from computed tomography images. Intell.-Based Med..

[46] Kim D., Lee S., Park J. (2025). Improved Consistency of Lung Nodule Categorization in CT Scans with Heterogeneous Slice Thickness by Deep Learning-Based 3D Super-Resolution. Diagnostics.

[47] Yang J., Zhang L., Wang D. (2016). Uncertainty analysis of quantitative imaging features extracted from contrast-enhanced CT in lung tumors. Comput. Med. Imaging Graph..

[48] Sadremomtaz A., Zadnorouzi M. (2024). Improving the quality of pulmonary nodules segmentation using the new proposed U-Net neural network. Intell.-Based Med..

[49] Hu T., Lan Y., Zhang Y. (2024). A Lung Nodule Segmentation Model Based on the Transformer with Multiple Thresholds and Coordinate Attention. Sci. Rep..

[50] A.R. B., R.S. V.K., S.S. K. (2023). LCD-Capsule Network for the Detection and Classification of Lung Cancer on Computed Tomography Images. Multimed. Tools Appl..

[51] Singh V.K., Patel A.K. Transfer Learning Ensemble Model for Lung Nodule Classification. Proceedings of the 2024 International Conference on Integrated Circuits, Communication, and Computing Systems (ICIC3S).

[52] Li G., Li Z., Hu X. (2020). Study on the Detection of Pulmonary Nodules in CT Images Based on Deep Learning. IEEE Access.

[53] Ren Y., Wang Z., Zhou Y. (2020). A manifold learning regularization approach to enhance 3D CT image-based lung nodule classification. Neural Comput. Appl..

[54] de Sousa Costa R.W., da Silva G.L.F., de Carvalho Filho A.O., Silva A.C., de Paiva A.C., Gattass M. (2018). Classification of malignant and benign lung nodules using taxonomic diversity index and phylogenetic distance. Med. Biol. Eng. Comput..

[55] Khan M.A., Sharif M., Akram T., Raza M. (2019). Hand-Crafted and Deep Convolutional Neural Network Features Fusion and Selection Strategy: An Application to Intelligent Human Action Recognition. Appl. Soft Comput..

[56] Shatnawi M., Al-Abweh M., Nofal M. (2025). Deep Learning-Based Approach to Diagnose Lung Cancer Using CT-Scan Images. Intell.-Based Med..

[57] Liu D., Liu F., Tie Y., Qi L., Wang F. (2022). Res-Trans Networks for Lung Nodule Classification. Int. J. Comput. Assist. Radiol. Surg..

[58] Crasta L.J., Neema R., Pais A.R. (2024). A novel Deep Learning architecture for lung cancer detection and diagnosis from Computed Tomography image analysis. Healthc. Anal..

[59] Nai Y.H., Teo B.K., Tan C.H., O’Donnell S., Steinberg I., Levin C.S. (2021). Validation of low-dose lung cancer PET-CT protocol and PET image improvement using machine learning. Phys. Med..

[60] Acharya U., Kumar S. (2021). Genetic Algorithm-based Adaptive Histogram Equalization (GAAHE) technique for for medical image enhancement. Optik.

[61] Alsheikhy A.A., Said Y., Shawky M. (2023). A CAD System for Lung Cancer Detection Using Hybrid Deep Learning Techniques. Diagnostics.

[62] Chen Y., Wang Y., Hu F., Feng L., Zhou T., Zheng C. (2021). LDNNET: Towards Robust Classification of Lung Nodule and Cancer Using Lung Dense Neural Network. IEEE Access.

[63] Zuo H. CDAE-C: A Fully Convolutional Denoising Auto-Encoder with 2.5D Convolutional Classifier. Proceedings of the 2022 IEEE Conference on Telecommunications, Optics and Computer Science (TOCS).

[64] Li S., Zhao J., Han X. (2023). TPFR-Net: U-shaped model for lung nodule segmentation based on transformer pooling and dual attention. Comput. Biol. Med..

[65] Yadav A., Welland S., Hoffman J.M., Kim G.H.J., Brown M.S., Prosper A.E., Aberle D.R., McNitt-Gray M.F., Hsu W. (2023). A Comparative Analysis of Image Harmonization Techniques in Mitigating Differences in CT Acquisition and Reconstruction. Phys. Med. Biol..

[66] Dawood H., Dawood A., Guo R. (2025). Attention-guided CenterNet deep learning approach for lung cancer detection. Comput. Biol. Med..

[67] Sun Y., Liu H., Zhao Z. (2024). Performance and application of the total-body PET/CT scanner: A literature review. EJNMMI Res..

[68] Jiang Y., Gong X., Liu D., Cheng Y., Fang C., Shen X., Yang J., Zhou P., Wang Z. (2021). EnlightenGAN: Deep Light Enhancement without Paired Supervision. IEEE Trans. Image Process..

[69] Schmitz H., Kurz C., Kamp F., Klement R., Belka C., Landry G. (2023). ScatterNet for projection-based 4D cone-beam CT intensity correction of lung cancer patients. Phys. Imaging Radiat. Oncol..

[70] Tian Q., Li Z., Chen G. (2022). Image quality improvement in low-dose chest CT with deep learning image reconstruction. J. Appl. Clin. Med. Phys..

[71] D’hondt L., Mollet P., Vandenberghe S. (2024). Impact of deep learning image reconstruction on volumetric accuracy and image quality of pulmonary nodules with different morphologies in low-dose CT. Cancer Imaging.

[72] Santos J.C., Alexandre H.T.P., Santos M.S., Abreu P.H. (2025). The Role of Deep Learning in Medical Image Inpainting: A Systematic Review. ACM Trans. Comput. Healthc..

[73] Lin X., Wang J., Wang Q., Yang Q., Li Y. (2025). Multi-window uncertainty-guided network for lung nodule CT segmentation. Alex. Eng. J..

[74] Wu Z., Li X., Zuo J. (2023). RAD-UNet: Research on an Improved Lung Nodule Semantic Segmentation Algorithm Based on Deep Learning. Front. Oncol..

[75] Bruntha P.M., Pandian S.I.A., Sagayam K.M. (2022). Lung_PAYNet: A Pyramidal Attention-Based Deep Learning Network for Lung Nodule Segmentation. Sci. Rep..

[76] Gayathri T., Kumar S., Singh R. Segmentation and Classification of Lung Tumor Analysis using LU-Net with BBH Optimizer. Proceedings of the 2024 14th International Conference on Cloud Computing, Data Science & Engineering (Confluence).

[77] Poonkodi S., Kanchana M. (2024). Lung cancer segmentation using modified mayfly optimization and PSO. Multimed. Tools Appl..

[78] Tang M., Xia J., Xu Z. 3D V-Net based segmentation for LUNA16 challenge. Proceedings of the MICCAI Lung Nodule Challenge.

[79] Ma Q., Liu S., Tang J. (2024). Improved V-Net lung nodule segmentation model based on pixel threshold separation and attention mechanism. Comput. Biol. Med..

[80] Heinrich M.P., Stille M., Buzug T.M. (2018). Residual U-Net Convolutional Neural Network Architecture for Low-Dose CT Denoising. Curr. Dir. Biomed. Eng..

[81] Pfeffer M.A., Ling S.H. (2022). Evolving Optimised Convolutional Neural Networks for Lung Cancer Classification. Signals.

[82] Faizi M.K., Qiang Y., Wei Y., Qiao Y., Zhao J., Aftab R., Ur Rehman Z. (2025). Deep Learning-Based Lung Cancer Classification of CT Images. BMC Cancer.

[83] Faruqui N., Yousuf M.A., Whaiduzzaman M., Azad A.K.M., Barros A., Moni M.A. (2021). LungNet: A Hybrid Deep-CNN Model for Lung Cancer Diagnosis Using CT and Wearable Sensor-Based Medical IoT Data. Comput. Biol. Med..

[84] Mienye I.D., Swart T.G., Obaido G., Jordan M., Ilono P. (2025). Deep Convolutional Neural Networks in Medical Image Analysis: A Review. Information.

[85] Cao H., Liu H., Song E., Ma G., Xu X., Jin R., Liu T., Hung C.-C. (2020). A Two-Stage Convolutional Neural Networks for Lung Nodule Detection. IEEE J. Biomed. Health Inform..

[86] Çay T. (2025). Lung Cancer Diagnosis with GAN-Supported Deep Learning Models. Biomed. Mater. Eng..

[87] Angel Mary A., Thanammal K.K. (2023). Lung Cancer Detection via Deep Learning-Based Pyramid Network with Honey Badger Algorithm. Meas. Sens..

[88] Qiao X., Zhang Y., Ma L. (2023). Ensemble framework based on attributes and deep features for benign–malignant classification of lung nodules. Pattern Recognit. Lett..

[89] Riquelme D., Akhloufi M.A. (2020). Deep Learning for Lung Cancer Nodules Detection and Classification in CT Scans. AI.

[90] Nemoto M., Ushifusa K., Kimura Y., Nagaoka T., Yamada T., Yoshikawa T. (2023). Unsupervised Feature Extraction for Various Computer-Aided Diagnosis Using Multiple Convolutional Autoencoders and 2.5-Dimensional Local Image Analysis. Appl. Sci..

[91] Sultana Z., Foysal M., Islam S., Foysal A.A. Lung cancer detection and classification from chest CT images using an ensemble deep learning approach. Proceedings of the 2024 6th International Conference on Electrical Engineering and Information & Communication Technology (ICEEICT).

[92] Zahari R., Cox J., Obara B. Quantifying the Uncertainty in 3D CT Lung Cancer Images Classification. Proceedings of the 2023 IEEE 13th International Conference on Pattern Recognition Systems (ICPRS).

[93] Jiang B., Li N., Shi X., Zhang S., Li J., de Bock G., Vliegenthart R. (2022). Deep Learning Reconstruction Shows Better Lung Nodule Detection for Ultra–Low-Dose Chest CT. Radiology.

[94] Wang T.W., van Buchem M.G.F., Cornelissen L.J., Kuijk S.M.J.v., Beets-Tan R.G.H. (2024). Systematic review and meta-analysis of deep learning applications in computed tomography lung cancer segmentation. Radiother. Oncol..

[95] Kesuma L.I., Octavia P., Sari P., Batubara G.M.C., Karina K. (2023). Combination of Gamma Correction and Vision Transformer in Lung Infection Classification on CT-Scan Images. J. Electr. Eng. Electron. Inform..

[96] Venkatesh C., Kumar S., Singh R. (2024). A hybrid model for lung cancer prediction using patch processing and deep learning on CT images. Multimed. Tools Appl..

[97] Murthy N.N., Thippeswamy K. (2025). Fuzzy-ER Net: Fuzzy-based efficient residual network-based lung cancer classification. Comput. Electr. Eng..

